# Epigenetic Alterations in Colitis-Associated Colorectal Cancer

**DOI:** 10.3390/epigenomes10010004

**Published:** 2026-01-16

**Authors:** Nisha Ganesh, William M. Grady, Andrew M. Kaz

**Affiliations:** 1 Division of Gastroenterology, School of Medicine, University of Washington, Seattle, WA 98105, USA; nishagan@uw.edu (N.G.); wgrady@fredhutch.org (W.M.G.); 2 Translational Science and Therapeutics Division, Fred Hutchinson Cancer Center, Seattle, WA 98109, USA; 3 Public Health Sciences Division, Fred Hutchinson Cancer Center, Seattle, WA 98109, USA; 4 Clinical Research Division, Fred Hutchinson Cancer Center, Seattle, WA 98109, USA; 5 GI Section, Hospital and Specialty Medicine, VA Puget Sound Healthcare System, Seattle, WA 98108, USA

**Keywords:** colitis-associated colorectal cancer, inflammatory bowel disease, Crohn’s disease, ulcerative colitis, epigenetics, DNA methylation, histone modifications, micro-RNA, biomarkers

## Abstract

Colitis-associated colorectal cancer (CAC) represents a distinct subtype of colorectal malignancy that arises in the setting of chronic inflammatory bowel disease (IBD). Unlike sporadic colorectal cancer, CAC develops through inflammation-driven molecular pathways, in which epigenetic alterations play a pivotal role in tumor initiation and progression. This review highlights the major epigenetic mechanisms implicated in CAC, including DNA methylation, histone modifications, and microRNA (miRNA) dysregulation. Aberrant DNA methylation patterns, such as promoter hypermethylation of tumor suppressor genes and global hypomethylation, contribute to genomic instability and altered gene expression. In parallel, inflammation-induced changes in histone configuration modulate chromatin accessibility and transcriptional activity of key oncogenic and tumor-suppressive pathways. Furthermore, deregulated miRNAs influence multiple aspects of CAC pathogenesis by targeting genes involved in inflammation and tumor progression. Understanding these epigenetic processes provides valuable insights into the development of colorectal malignancy and identifies potential biomarkers for early detection and intervention in colitis-associated colorectal cancer.

## 1. Introduction

Inflammatory bowel disease (IBD) is a chronic, relapsing inflammatory condition of the gastrointestinal tract which affects millions worldwide. IBD, which includes both Crohn’s disease (CD) and ulcerative colitis (UC), is characterized by dysregulated immune responses and chronic mucosal inflammation. The pathophysiology is thought to be related to a complex interplay between genetic predisposition, environmental triggers, dysbiosis of the gut microbiota, immune dysregulation, and defective mucosal barrier function [1].

One of the most serious long-term complications of IBD is the development of colitis-associated colorectal cancer (CAC) which arises in the context of persistent mucosal inflammation. Chronic inflammation promotes cancer formation through a variety of cellular and molecular mechanisms, including injury to epithelial cells, leading to oxidative stress and DNA damage, thus driving tumorigenesis through the activation of oncogenic pathways [2]. Overall, individuals diagnosed with IBD carry up to two times the risk of colorectal cancer relative to the general population [3].

There are several major risk factors found to increase the risk of CAC, including duration of disease, extent of inflammation, cumulative inflammatory burden, and family history of first-degree relatives with colorectal cancer (CRC). Primary sclerosing cholangitis (PSC) also confers about a five times higher risk of colorectal cancer [1]. Screening guidelines from major gastroenterology societies recommend the initiation of CRC screening with colonoscopy, ideally incorporating chromoendoscopy, at 8–10 years following symptom onset for pancolitis, or at the time of PSC diagnosis [4]. Surveillance intervals in individuals with IBD vary based on risk. High risk disease, which includes those with PSC, prior dysplasia, a family history of CRC, and ongoing active inflammation benefit from yearly colonoscopy, while individuals with lower-risk disease, including limited colitis or inactive disease, require screening every three years [1,3].

With regard to the molecular alterations that mediate CAC formation, epigenetic alterations, including aberrant DNA methylation, histone modifications, and non-coding RNAs, have emerged in recent years as contributors to the initiation and progression of CAC. These alterations occur early in the neoplastic process, offering the potential to be used as biomarkers for early diagnosis, risk stratification, and therapeutic targeting. Alterations can then accumulate over years of relapsing colitis, progressively altering gene expression that modulates barrier function, DNA repair, immune responsiveness, and cell proliferation. As a result, epigenetic changes can not only mark the early stages of neoplastic transformation, but also actively participate in the development of CAC. These epigenetic signatures are distinct from those seen in sporadic colorectal cancer, with IBD-associated tumors exhibiting unique methylation patterns, histone modifications, and non-coding RNA profiles [5,6]. This review explores the current understanding of epigenetic mechanisms implicated in CAC pathogenesis and summarizes the major epigenetic alterations that have emerged as potential biomarkers for CAC.

## 2. Pathogenesis of CAC

Chronic inflammation creates a fertile environment for cancer development throughout the GI tract through an inflammation-induced dysplasia-to-carcinoma sequence. Examples of this include chronic gastritis associated with *Helicobacter pylori* infection leading to gastric adenocarcinoma or MALT lymphoma, gastrointestinal reflux leading to Barrett’s esophagus and the development of esophageal adenocarcinoma, and small bowel inflammation related to celiac disease increasing the risk of T-cell lymphoma or small bowel adenocarcinoma [7].

In the colon, CAC arises as a long-term complication of chronic IBD through an inflammation-driven pathway characterized by prolonged immune activation, oxidative stress, and epithelial regeneration. First, activation of inflammatory signaling pathways central to the pathogenesis of CAC can lead to increased transcription of tumor-promoting genes or pathways, such as the NF-kb and STAT3 signaling pathways [8]. Chronic inflammation induces repeated cycles of epithelial injury and repair, which leads to increased cell turnover and accumulation of DNA damage [9]. Inflammatory cells can also produce reactive oxygen species which promote oxidative DNA damage, and, over time, this damage is believed to overwhelm repair mechanisms and foster genomic instability [7].

Furthermore, the disruption of the intestinal epithelial barrier can lead to dysbiosis of the gut microbiome and contribute to carcinogenesis [10]. Patients with IBD exhibit reduced colonic microbial diversity, often with increased density of pro-inflammatory species and reduced density of protective bacterial species [11]. Some microbes can drive inflammation, produce toxins that induce DNA damage, alter host gene expression, and form metabolites that promote tumorigenesis [11]. Additionally, there is emerging evidence that bacterial biofilms can foster a microenvironment that promotes the development of dysplasia [12].

There are several molecular alterations that distinguish CAC and sporadic CRC. In inflammation-associated colitis, mutations in *TP53* generally occur early and are followed by additional gene alterations such as mutations in *KRAS* and *CTNNB1* (gene for β-catenin), as opposed to sporadic CRC in which early *APC* mutations are common [9]. Both CAC and sporadic CRC progress along the typical dysplasia-carcinoma sequence, starting with low-grade to high-grade dysplasia and eventually leading to invasive carcinoma. When detected by endoscopy, sporadic tumors are more likely to appear as polypoid, exophytic masses that are often well-demarcated and develop in the setting of otherwise normal-appearing mucosa. In comparison, dysplasia in CAC often appears flat and multifocal, and is frequently difficult to appreciate visually. CAC typically occurs in areas of high inflammatory burden, which can make routine surveillance and early detection more challenging [8]. CAC is more often poorly differentiated histologically, with mucinous and signet ring features, as well as irregular crypt architecture. CAC is also likely to demonstrate intramucosal spread and indistinct tumor margins with invasive features [9]. This contrasts with sporadic CRC, which is more often well to moderately differentiated. Given the important implications of early diagnosis and differentiation between IBD-CRC and sporadic CRC, there is a current need to develop clinically useful biomarkers for early detection of CAC.

## 3. Epigenetic Mechanisms in IBD-CRC

Inflammation can not only directly promote CAC but can also drive epigenetic modifications which can silence tumor suppressor genes and activate oncogenes. Epithelial cells are the primary site of inflammation-induced epigenetic changes, but immune cells and stromal cells also contribute to the remodeling of the tumor microenvironment through cytokine secretion and direct epigenetic modifications [13,14]. These epigenetic alterations then perpetuate inflammation by silencing anti-inflammatory genes, enhancing pro-inflammatory cytokine production, and altering epithelial barrier function [13,14].

Chronic inflammation leads to aberrant DNA methylation patterns that contribute to carcinogenesis by altering the expression of tumor suppressor genes, oncogenes, and genes involved in immune regulation and DNA repair [14,15,16]. DNA methylation typically occurs within CpG islands located in gene promoter regions, and the addition of a methyl group is catalyzed by DNA methyltransferases (DNMTs) [15,17]. In carcinogenesis, methylation abnormalities that are believed to mediate the cancer formation process generally include either promoter hypermethylation of tumor suppressor genes leading to transcriptional silencing, or global hypomethylation, which may result in genomic instability and the activation of oncogenic elements [15].

Another epigenetic mechanism involved in inflammation includes histone modification. Inflammatory signaling can regulate chromatin accessibility, thus impacting the silencing of anti-inflammatory and tumor suppressor genes [18]. Chronic inflammation also dysregulates the expression of miRNAs, small molecules that post-transcriptionally regulate gene expression [6]. Chronic inflammation leads to a microenvironment that can influence the expression of DNMTs through oxidative stress and the promotion of pro-inflammatory cytokines [15]. Genes involved in the cell cycle and DNA mismatch repair are often hypermethylated in CAC, leading to inactivation [15,19]. This includes promoter hypermethylation of genes such as *p16*, *p14*, *APC1A*, *APC2*, *SFRP1*, and *SFRP2* [20,21]. Given that these DNA methylation changes often precede morphological dysplasia, they could serve as possible biomarkers for early detection, risk stratification, and disease monitoring in IBD patients.

Inflammatory cytokines such as TNF-α, and IL-6 regulate the activity of DNMTs and histone deacetylase and methyltransferase in CAC by activating key transcriptional pathways which drive epigenetic reprogramming [13,14]. Cell line studies have been utilized to investigate this relationship between pro-inflammatory signals and alternations in DNA methylation and chromatin remodeling. Li et al. examined cell lines in colorectal tissue to evaluate the role of the inflammatory cytokine interleukin 6 (IL-6) signaling in colon cancer cells. IL-6 was found to be linked to DNMT1-dependent hypermethylation of the suppressor of cytokine signaling 3 (*SOCS3*) promoter, which enhances oncogenic STAT3 activity [22]. Further, IBD-related micro RNAs may affect expression of cell-cycle and chromatin-linked regulators. In cell line studies, miRNA let-7e and miR-17 was found to reduce the expression of the TP53 regulator E2F1 and miR-122. miR-17 modulates the expression of multiple transcription factors in the TP53 pathway, and miR-122 targets cell cycle regulator cyclin G [23]. This data supports a mechanistic bridge from inflammation-driven markers to epigenetic change.

## 4. Key Epigenetic Biomarkers in CAC

Colitis-associated colorectal cancers develop against a backdrop of chronic inflammation, resulting in multiple unique molecular features. In recent years, these epigenetic markers have emerged as promising tools for early detection, prognosis, and therapeutic targeting in CAC. These markers, which include DNA methylation alterations, histone modifications, and non-coding miRNAs, have the potential to improve clinical outcomes through identification of individuals with dysplasia at risk for progression versus non-progression. Table 1 summarizes recent research into DNA methylation markers that have potential for further study in CAC.

### 4.1. DNA Methylation

One of the most well-studied epigenetic processes that can lead to the development of CAC is aberrant DNA methylation. Several studies have investigated specific DNA methylation markers and their significance in the development of CAC. For example, a study by Li et al. identified 811 genes that were hypermethylated at various time points during the initiation and progression of colorectal cancer using DNA methylation sequencing. Genes that were hypermethylated and downregulated in the progression to CAC included those involved in the MAPK signaling pathway, KIT receptor signaling pathways, apoptosis pathways, and EGF/EGFR signaling pathways [24]. Additionally, low doses of DNA methyltransferase inhibitors administered to mice with CAC were found to exert antitumor effects, supporting the concept that DNA methylation plays a critical role in carcinogenesis [24].

Additional individual genes that have been shown to be highly methylated in colitis-associated cancer include *RUNX3*, *MINT1*, *MYOD*, and *CDKN2A* (also known as *p16* exon 1), in addition to the promoter regions of *EYA4* and *ESR* [25]. One study utilized mouse models to identify peripheral circulating factors, including circulating plasma DNA levels and Alu methylation levels that correlated with the progression to CRC in the setting of chronic colitis [26]. Alu methylation has been shown to increase genomic instability and promote tumorigenesis [27]. Further study of aberrant DNA methylation in the colon, as well as in circulating leukocytes, appears to have the potential to lead to new diagnostic tests and therapies for prevention and/or treatment of CAC.

DNA methylation at CpG sites is associated with CAC through the accumulation of aberrant methylation patterns in inflamed colonic mucosa, leading to silencing of tumor suppressor genes. As the disease duration and severity increase, progressive hypermethylation of promoter CpG islands predisposes to neoplastic transformation [15,24,28]. CpG sites have been analyzed to evaluate genes whose methylation patterns appear to correlate with the development of CAC. For example, the hypermethylation of specific CpG sites of the genes *MICAL3* (microtubule associated monooxygenase), *MAD1L1* (Mitotic Spindle Assembly checkpoint protein), and *METTL22* (Methyltransferase-like protein) at defined levels have been found to be associated with UC-CRC. The ENDCAP-C trial, a multicenter study to assess the role of molecular markers in improving the detection of dysplasia, identified the methylation of a five-marker panel (*SFRP2*, *SFRP4*, *WIF1*, *APC1A*, *APC2*) that accurately detected ulcerative colitis associated dysplasia. This six-center UK study utilized a predictive model for UC associated dysplasia, and found that this marker panel identified pre-cancerous changes and invasive neoplasia in colonic tissue with an area under the curve (AUC) of 0.83 (95% CI 0.62–0.73) [29].

DNA methylation changes in CAC have also been compared to those found in Lynch syndrome cancers, with a recent study showing a high expression of *CD274* in CAC, similar to that in Lynch-syndrome-associated tumors [28]. Lastly, promoter methylation of *MSH6* and *TUMP3* have also been found to correlate with IBD-related dysplasia and cancer [30].

Persistent inflammation in IBD can lead to widespread epigenetic changes known as epigenetic field defects which serve as precursor states for neoplasia. In this setting, an increase in inflammatory mediators leads to activation of DNMTs which cause aberrant DNA methylation patterns. Specifically, DNA methylation of inflammatory marker genes can further attenuate the development of CAC [31]. For example, inflammation-associated genes, including *NTSR1* and CpG Island Methylator Phenotype (CIMP) markers, were found to have higher methylation levels in normal mucosa from CAC compared to normal mucosa in Lynch syndrome patients [28].

**Table 1:** Summary of recently identified DNA methylation markers associated with CAC.

Biomarker	Sample Type	Primary Result	Major Finding	Reference
*Alu* methylation level	Peripheral blood, mouse model	Methylation level of two restriction enzyme cutting sites significantly decreased (*p* < 0.05) in colitis associated cancer group versus normal control	Decrease in methylation level in UC-CRC compared to normal controls	[26]
ANPEP gene products	Colonic tissue	Significantly fewer UC-CRC tissue had brush border and tight junction expression of ANPEP compared to tissue adjacent to UC-CRC (*p* = 0.001) and normal controls (*p* < 0.0001). ANPEP gene products demonstrate an inverse correlation with methylation levels in CAC.	Downregulation in UC brush border in UC-CRC	[32]
*MICAL3*, *MAD1L1*, *METTL22*	Colonic tissue	Identified three methylation sites relevant to distinguishing CAC from sporadic CRC and defined methylation limit values. Combination of *MICAL3*, *MAD1L1*, and *METTL22* methylation sites allowed for correct assignment to CAC or sporadic CRC in 94.5% of cases	Defined methylation levels at three methylation sites can distinguish between CAC and sporadic CRC	[33]
*SFRP2*, *SFRP4*, *WIF1*, *APC1A*, *APC2*	Colonic tissue	Panel was accurate in detecting pre-cancerous and invasive neoplasia (AUC = 0.83; 95% CI 0.79, 0.88), and dysplasia (AUC = 0.88; (0.84, 0.91)).	Multiplex methylation marker panel accurate for UC-CRC detection	[29]
*MSH6, TIMP3*	Colonic tissue	Promoter methylation was significantly more common in IBD-related dysplasia or cancer in *MSH6* (5/10 vs. 1/31, *p* = 0.002), and *TIMP3* (4/9 vs. 1/25, *p* = 0.012) versus non-IBD related dysplasia or cancer samples	Promoter methylation associated with IBD related dysplasia/cancer	[30]
*NTSR1, CD274*	Colonic tissue	Hypermethylation frequency of *NTSR1* in CAC versus normal tissue (*p* = 0.037) Half of the CACs (16/31, 52%) expressed CD274-positive immune cells, whereas tumor cells were CD274-negative.	Increased level of methylation in CAC, similar to Lynch syndrome	[28]

Abbreviations: UC, ulcerative colitis; CRC, colorectal cancer; IBD, inflammatory bowel disease; ANPEP, Alanyl aminopeptidase; *MICAL3*, microtubule associated monooxygenase; *MAD1L1*, Mitotic Spindle Assembly checkpoint protein; *METTL22*, Methyltransferase-like protein; *SFRP*, Secreted Frizzled-Related Protein; *WIF1*, Wnt inhibitory factor 1; *APC*, adenomatous polyposis coli; *MSH6*, mutS homolog 6; *TIMP3*, Tissue Inhibitor of Metalloproteinase 3; *NTSR1*, Neurotensin Receptor 1.

Research into CAC has suggested ways in which the gut microbiome alterations seen in those with IBD may be interconnected with DNA methylation and the development of CRC in IBD. Chronic inflammation alters the gut microbial landscape, and in turn microbial metabolites and host-microbiome interactions can drive epigenetic changes that promote carcinogenesis [11]. For example, enrichment of *Fusobacterium nucleatum* has been associated with colorectal cancer in individuals with the CpG island methylator phenotype (CIMP). The mechanism of this is unclear, but it may be that the gut microbiota plays a role in the regulation of DNMTs [10]. Previous research has investigated the epigenetic regulation of colon cancer related genes by the *Lactobacillus acidophilus* strain NCK2025 and found that both in vivo and in vitro this strain enhanced the expression of tumor suppressor genes [34]. Further research into the relationship between gut microbiota and IBD related CRC is indicated.

### 4.2. Histone Modification

Histone modifications represent another key epigenetic process that can contribute to the development of CAC, as these molecular alterations can affect chromatin structure and gene expression. Histones are a family of proteins that package and organize DNA into structural units known as nucleosomes. DNA wraps around a histone core to form higher-order chromatin structures [35]. These histones affect gene expression by regulating access to DNA, as tightly packed heterochromatin is transcriptionally silent and loosely packed euchromatin is transcriptionally active. Histones can be modified after translation by several mechanisms, including acetylation, methylation, and phosphorylation, which may either activate or repress gene expression. Histones may also be impacted directly by methylated DNA, which recruits histone-modifying enzymes such as histone deacetylases and histone methyltransferases [36].

Multiple histone-modifying enzymes have been studied regarding their involvement in the development of dysplasia and CAC, including HDAC1/2, EZH2, and SETDB1 [37,38]. A study by Carvalho et al. retrospectively examined tissue from 207 CRC patients and evaluated the expression of histone-modifying enzymes EZH2 and SETDB1 (histone methyltransferases), and LSD-1 (histone demethylase), which are associated with trimethylation of lysine 27 and lysine 9 on histone H3 (H3K27me3 and H3K9me3, respectively). In the CRC samples, there was increased expression of LSD-1 and EZH2, and decreased expression of SETDB1 [37]. Additional research has implicated histone deacetylase enzymes (HDAC 1, HDAC 2) in the development of CRC possibly related to their effects on increasing tumor proliferation and invasion [38].

Clinical trials have explored histone-modifying enzymes as potential therapeutic targets for management of IBD and colorectal cancer, although with limited results [39]. For example, the histone deacetylase inhibitor entinostat induces the ligation of Natural Killer (NK) cell activating receptors, augmenting the activity of cytotoxic NK cells against neoplastic cells [40]. However, a phase I clinical trial of regorafenib, hydroxychloroquine, and entinostat showed poor tolerance and similar efficacy to regorafenib alone [41]. Similarly, the EMERGE trial evaluated the effectiveness of domatinostat, a histone deacetylase inhibitor, with the anti-PD-L1 antibody avelumab in the treatment of previously treated esophageal adenocarcinoma and colorectal cancer. While the responses in the esophageal adenocarcinoma group met criteria to progress to a stage II trial, the colorectal cancer group showed insufficient efficacy to progress to the next phase [42]. This remains an area of active study with ongoing clinical trials. For example, there is an active trial (NCT05694936) using combination therapy of the histone deacetylase inhibitor sodium valproate with the standard of care anti-EGFR monoclonal antibody in the first-line treatment of individuals diagnosed with metastatic colon cancer.

### 4.3. MicroRNA Alterations

MicroRNAs (miRNA) may also play a role in the progression of CAC, presumably related to their role mediating the crosstalk between inflammatory pathways, immune signaling, and tumorigenesis. miRNA are small, non-coding RNAs that regulate gene expression post-transcriptionally by binding complimentary sequences to target mRNA, leading to degradation or translation inhibition [43]. Cell-free or circulating miRNA serve as promising biomarkers for the early detection of CAC, as they are released from inflamed or dysplastic tissue and remain stable within biofluids. The aberrant expression of these microRNAs reflects underlying molecular events in chronic inflammation and carcinogenesis [44]. For example, miR-21 acts on tumor suppressor genes such as *PTEN* and is upregulated in IBD, likely also playing a role in the development of CAC [43]. Further, miRNA 21 is thought to impair the intestinal barrier function, leading to gut dysbiosis and progressive inflammation [45]. Other miRNAs that have been studied in the development of CAC include miR-155, miR-301a, miR-34b-5p, and miR-124a [43,46,47]. miR-34b-5p is a microRNA that targets c-MYC, which enhances tumorgenicity in CAC. An in vitro study found that the suppression of miR-34b-5p leads to enhanced c-MYC activity and increased tumor formation [46]. Another study utilized methylation-specific polymerase chain reaction (PCR) to quantify methylation levels of mi-RNAs and found that miR-124a genes are aberrantly methylated during carcinogenesis in individuals with UC, offering a potential biomarker to estimate the risk of CRC in these patients. A review by Bocchetti et al. summarized additional major upregulated (miR-18a, miR-19a, miR-21, miR-31, miR-155 and miR-214) and downregulated (miR-124, miR-193a-3p and miR-139-5p) microRNAs in CAC [6]. In general, miRNAs have the potential to serve as predictive biomarkers for IBD treatment and for the development of CAC. In the future, circulating microRNA panels may be developed and validated for noninvasive early detection and risk stratification in CAC [44].

Another emerging area of research examines the bidirectional interaction between microRNA and the gut microbiome in relation to the development of colorectal cancer [48,49]. In the inflamed gut, alterations in the composition of the microbiome drives aberrant expression of key miRNAs [49]. These dysregulated miRNAs may then modulate pathways, leading to the development of CAC. Emerging evidence has also suggested the presence of bidirectional communication, where host-derived miRNA can enter bacterial cells and influence gene expression, potentially reinforcing pro-tumorigenic microbiome states [50]. The miRNA-microbiome axis represents a bridge between chronic colitis and neoplastic progression with potential for further study in the early detection of CAC. Table 2 summarizes recent research into micro RNA markers.

## 5. Clinical Applications and Future Directions

Epigenetic biomarkers in CAC have the potential to change disease management by assisting in risk stratification and serving as diagnostic biomarkers and prognostic indicators. Non-invasive tests using blood or stool samples could be utilized to detect methylated DNA fragments for the risk stratification of IBD patients, allowing for the personalization of surveillance intervals. Furthermore, a longitudinal biomarker assessment during colonoscopy surveillance may be utilized to monitor neoplastic transformation by detection of the progressive methylation of tumor suppressor genes [57]. There also exists the possibility of dysplasia or cancer prevention using drugs that target aberrant epigenetic changes, such as DNMT inhibitors. A study from Li et al. investigated the role of low-dose DNMT inhibitors on mouse models of CAC. This group found that the treatment of mice with DNMT inhibitors resulted in fewer tumors and reduced tumor area, suggesting that CAC formation can be inhibited by targeting DNA methyltransferases [24]. Another study examined the use of the DNMT inhibitor decitabine to upregulate the Epstein-bar virus-induced gene 3 (*EBI3*), which results in the production of anti-inflammatory IL-35 through NFkB signaling [58]. This may represent a potential therapeutic target in individuals with ulcerative colitis, feasibly attenuating the formation of CAC. Future applications resulting from the analysis of DNA methylation patterns may include the development of biomarker panels to improve the risk stratification for colon dysplasia or CAC. Larger, prospective trials are still required to demonstrate the utility of predictive epigenetic panels.

## 6. Limitations

Epigenetic studies in CAC face several important limitations. Foremost is the marked heterogeneity of specimens, which include inflamed IBD biopsies, dysplastic lesions, and sporadic adenomas used as controls. These may contain variable proportions of neoplastic, inflamed, and normal mucosa, which can confound epigenetic profiling and interpretation [5,28]. Additionally, CAC arises in a background of chronic inflammation which itself induces widespread epigenetic alterations, making it difficult to separate cancer-specific changes from those drive by inflammation alone [15]. Many studies rely on small cohorts with limited demographic or clinical diversity, reducing statistical power and generalizability [29]. Furthermore, the scarcity of longitudinal prospective studies limits the validation of epigenetic biomarkers. The dynamic and reversible nature of epigenetic modifications complicates their use as stable biomarkers for early detection or risk stratification, and further longitudinal prospective studies are needed to clarify their relationship to neoplastic transformation [14,29].

## 7. Conclusions

Colitis-associated colorectal cancer remains one of the most serious complications of long-standing inflammatory bowel disease. This form of inflammation-associated cancer is driven by continuous cycles of epithelial injury and regeneration, which creates a microenvironment conducive to neoplastic transformation [9]. While the contribution of genetic mutations in CAC has been well-described, it is increasingly evident that epigenetic alterations play a critical role as well [59].

Key epigenetic alterations are summarized in this review, including DNA methylation of tumor related and inflammatory genes, effects of the gut microbiota, and modifications in histones and miRNA. These epigenetic changes may emerge early, often preceding histologic dysplasia, and can influence gene expression patterns that regulate cell proliferation, apoptosis, and immune signaling [59]. These alterations have the potential to play a role in early detection, diagnosis, risk stratification, and prognosis of CAC [60]. Additional studies of these and other epigenetic markers will be crucial to bring these advances to clinical practice.

## Figures and Tables

**Table 2:** Summary of major miRNA markers associated with development of CAC.

Biomarker	Sample Type	Major Finding	References
miR-21	Tissue, Serum	Upregulated in IBD and CAC, hypothesized to facilitate development of CAC	[43,44,51,52,53]
miR-15b	Tissue, Serum	Increased levels correlate with tumor progression, with high diagnostic accuracy for detection of early colonic lesion (AUC 1.00)	[44,53]
miR-29a	Tissue, Serum	Increased levels correlate to tumor progression, high diagnostic accuracy for detection of early colonic lesions (AUC 0.90)	[44,54]
miR-375	Plasma	Upregulated in CAC compared with active UC (*p* = 0.0061)	[55]
miR-34b-5p	Tissue	Suppressed in CAC tissue versus adjacent control tissue (*p* < 0.001)	[46]
miR-19a	Tissue	Upregulated in CAC. May promote development of CAC through stimulation of TNF-α	[45,56]

Abbreviations: UC, ulcerative colitis; IBD, inflammatory bowel disease; CAC, colitis-associated colorectal cancer; miR, microRNA; TNF, tumor necrosis factor; AUC, area under the curve.

## Data Availability

No new data were created or analyzed in this study. Data sharing is not applicable to this article.

## Abbreviations

The following abbreviations are used in this manuscript:

IBD	Inflammatory Bowel Disease
CAC	Colitis-Associated Colorectal Cancer
CD	Crohn’s disease
UC	Ulcerative Colitis
PSC	Primary Sclerosing Cholangitis
DNMT	DNA methyltransferase
NK	Natural Killer

## References

[1] Fatakhova K., Rajapakse R. (2024). From random to precise: Updated colon cancer screening and surveillance for inflammatory bowel disease. Transl. Gastroenterol. Hepatol..

[2] Laredo V., García-Mateo S., Martínez-Domínguez S.J., López de la Cruz J., Gargallo-Puyuelo C.J., Gomollón F. (2023). Risk of Cancer in Patients with Inflammatory Bowel Diseases and Keys for Patient Management. Cancers.

[3] Clarke W.T., Feuerstein J.D. (2019). Colorectal cancer surveillance in inflammatory bowel disease: Practice guidelines and recent developments. World J. Gastroenterol..

[4] Murthy S.K., Feuerstein J.D., Nguyen G.C., Velayos F.S. (2021). AGA Clinical Practice Update on Endoscopic Surveillance and Management of Colorectal Dysplasia in Inflammatory Bowel Diseases: Expert Review. Gastroenterology.

[5] Rajamäki K., Taira A., Katainen R., Välimäki N., Kuosmanen A., Plaketti R.M., Seppälä T.T., Ahtiainen M., Wirta E.V., Vartiainen E. (2021). Genetic and Epigenetic Characteristics of Inflammatory Bowel Disease-Associated Colorectal Cancer. Gastroenterology.

[6] Bocchetti M., Ferraro M.G., Ricciardiello F., Ottaiano A., Luce A., Cossu A.M., Scrima M., Leung W.Y., Abate M., Stiuso P. (2021). The Role of microRNAs in Development of Colitis-Associated Colorectal Cancer. Int. J. Mol. Sci..

[7] Vanoli A., Parente P., Fassan M., Mastracci L., Grillo F. (2023). Gut inflammation and tumorigenesis: Every site has a different tale to tell. Intern. Emerg. Med..

[8] Chen R., Lai L.A., Brentnall T.A., Pan S. (2016). Biomarkers for colitis-associated colorectal cancer. World J. Gastroenterol..

[9] Saraggi D., Fassan M., Mescoli C., Scarpa M., Valeri N., Michielan A., D’Incá R., Rugge M. (2017). The molecular landscape of colitis-associated carcinogenesis. Dig. Liver Dis..

[10] Gutierrez-Angulo M., Ayala-Madrigal M.L., Moreno-Ortiz J.M., Peregrina-Sandoval J., Garcia-Ayala F.D. (2023). Microbiota composition and its impact on DNA methylation in colorectal cancer. Front. Genet..

[11] Quaglio A.E.V., Grillo T.G., De Oliveira E.C.S., Di Stasi L.C., Sassaki L.Y. (2022). Gut microbiota, inflammatory bowel disease and colorectal cancer. World J. Gastroenterol..

[12] Muñiz Pedrogo D.A., Sears C.L., Melia J.M.P. (2024). Colorectal Cancer in Inflammatory Bowel Disease: A Review of the Role of Gut Microbiota and Bacterial Biofilms in Disease Pathogenesis. J. Crohns Colitis.

[13] Tan S.Y.X., Zhang J., Tee W.W. (2022). Epigenetic Regulation of Inflammatory Signaling and Inflammation-Induced Cancer. Front. Cell Dev. Biol..

[14] Triantaphyllopoulos K.A., Ragia N.D., Panagiotopoulou M.E., Sourlingas T.G. (2025). Integrating Inflammatory and Epigenetic Signatures in IBD-Associated Colorectal Carcinogenesis: Models, Mechanisms, and Clinical Implications. Int. J. Mol. Sci..

[15] Hartnett L., Egan L.J. (2012). Inflammation, DNA methylation and colitis-associated cancer. Carcinogenesis.

[16] Damiano O.M., Stevens A.J., Kenwright D.N., Seddon A.R. (2025). Chronic Inflammation to Cancer: The Impact of Oxidative Stress on DNA Methylation. Front. Biosci..

[17] Jair K.W., Bachman K.E., Suzuki H., Ting A.H., Rhee I., Yen R.W., Baylin S.B., Schuebel K.E. (2006). De novo CpG island methylation in human cancer cells. Cancer Res..

[18] Chen L., Luo Z., Zhao C., Li Q., Geng Y., Xiao Y., Chen M.K., Li L., Chen Z.X., Wu M. (2022). Dynamic Chromatin States Coupling with Key Transcription Factors in Colitis-Associated Colorectal Cancer. Adv. Sci..

[19] Fleisher A.S., Esteller M., Harpaz N., Leytin A., Rashid A., Xu Y., Liang J., Stine O.C., Yin J., Zou T.T. (2000). Microsatellite instability in inflammatory bowel disease-associated neoplastic lesions is associated with hypermethylation and diminished expression of the DNA mismatch repair gene, hMLH1. Cancer Res..

[20] Wang F.Y., Arisawa T., Tahara T., Takahama K., Watanabe M., Hirata I., Nakano H. (2008). Aberrant DNA methylation in ulcerative colitis without neoplasia. Hepatogastroenterology.

[21] Dhir M., Montgomery E.A., Glöckner S.C., Schuebel K.E., Hooker C.M., Herman J.G., Baylin S.B., Gearhart S.L., Ahuja N. (2008). Epigenetic regulation of WNT signaling pathway genes in inflammatory bowel disease (IBD) associated neoplasia. J. Gastrointest. Surg..

[22] Li Y., Deuring J., Peppelenbosch M.P., Kuipers E.J., de Haar C., van der Woude C.J. (2012). IL-6-induced DNMT1 activity mediates SOCS3 promoter hypermethylation in ulcerative colitis-related colorectal cancer. Carcinogenesis.

[23] Kanaan Z., Rai S.N., Eichenberger M.R., Barnes C., Dworkin A.M., Weller C., Cohen E., Roberts H., Keskey B., Petras R.E. (2012). Differential microRNA expression tracks neoplastic progression in inflammatory bowel disease-associated colorectal cancer. Hum. Mutat..

[24] Li J., Su X., Dai L., Chen N., Fang C., Dong Z., Fu J., Yu Y., Wang W., Zhang H. (2020). Temporal DNA methylation pattern and targeted therapy in colitis-associated cancer. Carcinogenesis.

[25] Emmett R.A., Davidson K.L., Gould N.J., Arasaradnam R.P. (2017). DNA methylation patterns in ulcerative colitis-associated cancer: A systematic review. Epigenomics.

[26] Bai X., Zhu Y., Pu W., Xiao L., Li K., Xing C., Jin Y. (2015). Circulating DNA and its methylation level in inflammatory bowel disease and related colon cancer. Int. J. Clin. Exp. Pathol..

[27] Ye D., Jiang D., Zhang X., Mao Y. (2020). Alu Methylation and Risk of Cancer: A Meta-analysis. Am. J. Med. Sci..

[28] Mäki-Nevala S., Ukwattage S., Wirta E.V., Ahtiainen M., Ristimäki A., Seppälä T.T., Lepistö A., Mecklin J.P., Peltomäki P. (2021). Immunoprofiles and DNA Methylation of Inflammatory Marker Genes in Ulcerative Colitis-Associated Colorectal Tumorigenesis. Biomolecules.

[29] Beggs A.D., Mehta S., Deeks J.J., James J.D., Caldwell G.M., Dilworth M.P., Stockton J.D., Blakeway D., Pestinger V., Vince A. (2019). Validation of epigenetic markers to identify colitis associated cancer: Results of module 1 of the ENDCAP-C study. eBioMedicine.

[30] Rosa I., Silva P., da Mata S., Magro F., Carneiro F., Peixoto A., Silva M., Sousa H.T., Roseira J., Parra J. (2020). Methylation patterns in dysplasia in inflammatory bowel disease patients. Scand. J. Gastroenterol..

[31] Cervena K., Siskova A., Buchler T., Vodicka P., Vymetalkova V. (2020). Methylation-Based Therapies for Colorectal Cancer. Cells.

[32] Pekow J., Hernandez K., Meckel K., Deng Z., Haider H.I., Khalil A., Zhang C., Talisila N., Siva S., Jasmine F. (2019). IBD-associated Colon Cancers Differ in DNA Methylation and Gene Expression Profiles Compared With Sporadic Colon Cancers. J. Crohns Colitis.

[33] Haumaier F., Dregelies T., Sterlacci W., Atreya R., Vieth M. (2024). Methylation Analysis of Colitis-Associated Colorectal Carcinomas. Discov. Med..

[34] Lightfoot Y.L., Yang T., Sahay B., Mohamadzadeh M. (2013). Targeting aberrant colon cancer-specific DNA methylation with lipoteichoic acid-deficient Lactobacillus acidophilus. Gut Microbes.

[35] Bannister A.J., Kouzarides T. (2011). Regulation of chromatin by histone modifications. Cell Res..

[36] Zhang Y., Sun Z., Jia J., Du T., Zhang N., Tang Y., Fang Y., Fang D. (2021). Overview of Histone Modification. Adv. Exp. Med. Biol..

[37] Carvalho S., Freitas M., Antunes L., Monteiro-Reis S., Vieira-Coimbra M., Tavares A., Paulino S., Videira J.F., Jerónimo C., Henrique R. (2018). Prognostic value of histone marks H3K27me3 and H3K9me3 and modifying enzymes EZH2, SETDB1 and LSD-1 in colorectal cancer. J. Cancer Res. Clin. Oncol..

[38] Gerçeker E., Boyacıoglu S.O., Kasap E., Baykan A., Yuceyar H., Yıldırım H., Ayhan S., Ellidokuz E., Korkmaz M. (2015). Never in mitosis gene A-related kinase 6 and aurora kinase A: New gene biomarkers in the conversion from ulcerative colitis to colorectal cancer. Oncol. Rep..

[39] Liang B., Wang Y., Xu J., Shao Y., Xing D. (2023). Unlocking the potential of targeting histone-modifying enzymes for treating IBD and CRC. Clin. Epigenetics.

[40] Zhu S., Denman C.J., Cobanoglu Z.S., Kiany S., Lau C.C., Gottschalk S.M., Hughes D.P., Kleinerman E.S., Lee D.A. (2015). The narrow-spectrum HDAC inhibitor entinostat enhances NKG2D expression without NK cell toxicity, leading to enhanced recognition of cancer cells. Pharm. Res..

[41] Karasic T.B., Brown T.J., Schneider C., Teitelbaum U.R., Reiss K.A., Mitchell T.C., Massa R.C., O’Hara M.H., DiCicco L., Garcia-Marcano L. (2022). Phase I Trial of Regorafenib, Hydroxychloroquine, and Entinostat in Metastatic Colorectal Cancer. Oncologist.

[42] Cartwright E., Slater S., Saffery C., Tran A., Turkes F., Smith G., Aresu M., Kohoutova D., Terlizzo M., Zhitkov O. (2024). Phase II trial of domatinostat (4SC-202) in combination with avelumab in patients with previously treated advanced mismatch repair proficient oesophagogastric and colorectal adenocarcinoma: EMERGE. ESMO Open.

[43] James J.P., Riis L.B., Malham M., Høgdall E., Langholz E., Nielsen B.S. (2020). MicroRNA Biomarkers in IBD-Differential Diagnosis and Prediction of Colitis-Associated Cancer. Int. J. Mol. Sci..

[44] El-Daly S.M., Morsy S.M., Medhat D., El-Bana M.A., Latif Y.A., Omara E.A., Awadallah J.R., Gamal-Eldeen A.M. (2019). The diagnostic efficacy of circulating miRNAs in monitoring the early development of colitis-induced colorectal cancer. J. Cell Biochem..

[45] Włodarczyk M., Maryńczak K., Burzyński J., Włodarczyk J., Basak J., Fichna J., Majsterek I., Ciesielski P., Spinelli A., Dziki Ł. (2025). The role of miRNAs in the pathogenesis, diagnosis, and treatment of colorectal cancer and colitis-associated cancer. Clin. Exp. Med..

[46] Yang C., Lu W., He H., Liu H. (2020). Inflammation and DNA Methylation-Dependent Down-Regulation of miR-34b-5p Mediates c-MYC Expression and CRL4(DCAF4) E3 Ligase Activity in Colitis-Associated Cancer. Am. J. Pathol..

[47] Ueda Y., Ando T., Nanjo S., Ushijima T., Sugiyama T. (2014). DNA methylation of microRNA-124a is a potential risk marker of colitis-associated cancer in patients with ulcerative colitis. Dig. Dis. Sci..

[48] Nikolaieva N., Sevcikova A., Omelka R., Martiniakova M., Mego M., Ciernikova S. (2022). Gut Microbiota-MicroRNA Interactions in Intestinal Homeostasis and Cancer Development. Microorganisms.

[49] Yuan C., Burns M.B., Subramanian S., Blekhman R. (2018). Interaction between Host MicroRNAs and the Gut Microbiota in Colorectal Cancer. mSystems.

[50] Yuan C., Steer C.J., Subramanian S. (2019). Host-MicroRNA-Microbiota Interactions in Colorectal Cancer. Genes.

[51] Ludwig K., Fassan M., Mescoli C., Pizzi M., Balistreri M., Albertoni L., Pucciarelli S., Scarpa M., Sturniolo G.C., Angriman I. (2013). PDCD4/miR-21 dysregulation in inflammatory bowel disease-associated carcinogenesis. Virchows Arch..

[52] Ahmed Hassan E., El-Din Abd El-Rehim A.S., Mohammed Kholef E.F., Abd-Elgwad Elsewify W. (2020). Potential role of plasma miR-21 and miR-92a in distinguishing between irritable bowel syndrome, ulcerative colitis, and colorectal cancer. Gastroenterol. Hepatol. Bed Bench.

[53] Sur D., Advani S., Braithwaite D. (2022). MicroRNA panels as diagnostic biomarkers for colorectal cancer: A systematic review and meta-analysis. Front. Med..

[54] Wang A., Deng S., Chen X., Yu C., Du Q., Wu Y., Chen G., Hu L., Hu C., Li Y. (2020). miR-29a-5p/STAT3 Positive Feedback Loop Regulates TETs in Colitis-Associated Colorectal Cancer. Inflamm. Bowel. Dis..

[55] Patel M., Verma A., Aslam I., Pringle H., Singh B. (2015). Novel plasma microRNA biomarkers for the identification of colitis-associated carcinoma. Lancet.

[56] Wang T., Xu X., Xu Q., Ren J., Shen S., Fan C., Hou Y. (2017). miR-19a promotes colitis-associated colorectal cancer by regulating tumor necrosis factor alpha-induced protein 3-NF-κB feedback loops. Oncogene.

[57] Gu X., Wei S., Lv X. (2024). Circulating tumor cells: From new biological insights to clinical practice. Signal Transduct. Target. Ther..

[58] Wetzel A., Scholtka B., Schumacher F., Rawel H., Geisendörfer B., Kleuser B. (2021). Epigenetic DNA Methylation of EBI3 Modulates Human Interleukin-35 Formation via NFkB Signaling: A Promising Therapeutic Option in Ulcerative Colitis. Int. J. Mol. Sci..

[59] Zhou R.W., Harpaz N., Itzkowitz S.H., Parsons R.E. (2023). Molecular mechanisms in colitis-associated colorectal cancer. Oncogenesis.

[60] Kritzinger J., Kotrri G., Lakatos P.L., Bessissow T., Wild G. (2025). The Role of Biomarkers in Surveillance of Ulcerative Colitis-Associated Colorectal Cancer: A Scoping Review. J. Clin. Med..

